# Assessing Climate Change Impacts on Distribution Dynamics of *Lysimachia Christinae* in China Through MaxEnt Modeling

**DOI:** 10.1002/ece3.71664

**Published:** 2025-06-24

**Authors:** Yangzhou Xiang, Yuan Li, Ying Liu, Yingying Yuan, Suhang Li, Qiong Yang, Jinxin Zhang

**Affiliations:** ^1^ School of Geography and Resources Guizhou Education University Guiyang China; ^2^ Grasslands and Sustainable Farming, Production Systems Unit Natural Resources Institute Finland Kuopio Finland; ^3^ School of Biological Sciences Guizhou Education University Guiyang China; ^4^ Institute of Ecological Conservation and Restoration Chinese Academy of Forestry/Grassland Research Center, National Forestry and Grassland Administration Beijing China

**Keywords:** climate change, ENMeval, geographical distribution, *Lysimachia christinae*, MaxEnt model

## Abstract

*Lysimachia christinae,* a regionally endemic medicinal plant in China, is crucial for ecosystems and traditional medicine. This study evaluates climate change impacts on the geographic spread of *L. christinae* by employing an optimized MaxEnt model based on 625 valid occurrence points and various climatic variables. The model was refined with ENMeval in R, selecting optimal feature combinations (FC) and regularization multipliers (RM). The model's predictive performance was evaluated via the AUC metric, and the distribution changes were analyzed across three Shared Socioeconomic Pathways (SSPs) spanning the 2050s, 2070s, and 2090s. The findings indicated that the refined MaxEnt model exhibited strong predictive performance, achieving an AUC of 0.904. The min temperature of coldest month (Bio6) and the standard deviation of temperature seasonality (Bio4) were identified as the principal climatic variables affecting the geographic range of *L. christinae* , contributing 68.7% and 20.2%, respectively, under current climatic conditions. Within the SSP1‐2.6 pathway, the viable habitat zone remained relatively stable, with retention rates of 86.78%, 86.13%, and 82.03% during the decades of the 2050s, 2070s, as well as 2090s. However, in the context of the SSP5‐8.5 pathway, the retention rate significantly decreased to 64.77% by the 2090s, indicating greater habitat instability and expansion needs. The research highlights the critical role of thermal variables in shaping *L. christinae* 's distribution and emphasizes the need for adaptive conservation strategies targeting stable or expanding habitats to ensure its long‐term survival amid climate change.

## Graphical abstract

This study employs an optimized MaxEnt model to assess the impact of climate change on the distribution dynamics of the regionally endemic medicinal plant Lysimachia christinae in China. The results indicate that the minimum temperature of the coldest month and the standard deviation of temperature seasonality are the primary climatic drivers affecting its geographic range. The results highlight significant changes in suitable habitats under different climate scenarios, emphasizing the need for adaptive conservation strategies for this species.

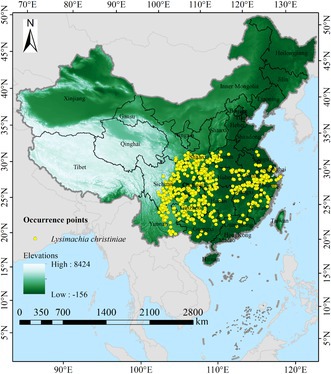

## 1 Introduction

*Lysimachia christinae* Hance belongs to the *Primulaceae* family and the genus *Lysimachia* (He et al. 2022). It is a perennial herbaceous plant widely distributed across China. This species is indispensable to ecosystems, playing a vital role in maintaining ecological balance and biodiversity. Additionally, it is valued in traditional Chinese medicine for treating hepatobiliary disorders, urolithiasis, and inflammatory conditions (Kim et al. 2020; Zhang et al. 2022; Zhou et al. 2023), occupying a unique and irreplaceable position. As global climate change becomes more pronounced, alterations in climate factors such as rising temperatures and shifting precipitation patterns are having increasingly profound influences on biodiversity (Harrison et al. 2024; Pereira et al. 2024). The distribution patterns of plants, as an integral part of biodiversity, face significant challenges. Many plants have experienced contractions, expansions, or shifts within their geographic ranges (Li et al. 2024; You et al. 2024), which not only affect their own survival but also trigger cascading influences on ecosystem stability and resilience. Therefore, examining how climate change influences plant distribution has risen to prominence as a central topic in current ecological studies. Given its status as a regionally characteristic medicinal herb, understanding the adaptive mechanisms and distribution shifts of *L. christinae* is crucial for preserving biodiversity in the context of global change.

The effects of climate change on biodiversity are diverse, impacting species viability, breeding success, and geographical distributions (Bellard et al. 2012; Pecl et al. 2017). As a specific example, climate change may force plants like *L. christinae* to adapt to novel conditions or shift their ranges to track suitable habitats. Such changes may impact the ecological niche of *L. christinae* , thereby affecting its function and status within ecosystems (Pugnaire et al. 2019). For instance, *L. christinae* may have to compete with other species for resources or face new threats from pests and diseases (He et al. 2022; Ma et al. 2021; Singh et al. 2023). Additionally, climate change may influence the reproductive and growth cycles of *L. christinae* , thereby affecting its population dynamics (Bogdziewicz 2022; Sato et al. 2024). These alterations pose challenges not only to *L. christinae* itself but also to other species that depend on it and to the entire ecosystem. Therefore, investigating the influence of climate change on the distribution and ecological niche of *L. christinae* is vital for the development of cultivation processes and conservation initiatives (Shi et al. 2023).

Although climate change impacts on plant distributions are well‐studied globally (Puchałka et al. 2021; Zheng et al. 2021; Zu et al. 2021), research on China's regionally endemic medicinal herb *L. christinae* remains limited. Current studies often focus on large‐scale, economically valuable trees or widely distributed herbaceous plants globally (Hartmann et al. 2022; Hosseini et al. 2024; Meng et al. 2021; Zhao et al. 2021), with less attention given to region‐specific medicinal herbs like *L. christinae* . Methodologically, many studies rely on traditional quadrat surveys combined with simple statistical analyses, lacking the application of more advanced modeling techniques. The maximum entropy (MaxEnt) modeling approach, a powerful species distribution prediction tool with unique advantages in forecasting how climate change affects species' geographic ranges (Elith et al. 2011; Merow et al. 2013; Phillips et al. 2006), has not yet been applied to the study of *L. christinae* . This tool can estimate the likely distribution areas of a species using existing distribution information and environmental factors, and it is capable of evaluating how various environmental factors impact species' geographic occurrence (Kaky et al. 2020). The application of such a model can provide more precise and in‐depth analysis for the study of distribution patterns for *L. christinae* and provide a scientific foundation for future conservation efforts and resource management.

This study seeks to utilize the MaxEnt model to precisely forecast shifts in the distribution dynamics of *L. christinae* in China amid climate change projections. Through examining the correlation between diverse climatic elements, like temperature and rainfall, and the geographic occurrence of *L. christinae* , the research aims to uncover its adaptive strategies to climate change, offering a scientific foundation for the preservation, sustainable use, and administration of associated ecosystems. The main questions addressed in this study include: How will the probable distribution areas of *L. christinae* in China shift amid climate change projections? Which climatic factors have the greatest influence on its geographic distribution dynamics? And how accurate is the MaxEnt model in predicting its distribution pattern? Exploring these questions will enhance our understanding of survival strategies for *L. christinae* within the framework of global environmental variability and its potential role in ecosystems, and offer a reference for the study of other plants within the same family and genus.

## 2 Materials and Methods

### 2.1 Gathering and Analyzing Species Geographical Presence Data

The foundational cartographic data for China were sourced from the authoritative Standard Map Service Platform administered by China's Ministry of Natural Resources (Approval No. GS [2023]2762; accessible at http://bzdt.ch.mnr.gov.cn). The spatial occurrence records for *L. christinae* across China were compiled through synthesizing multiple validated scientific repositories. Species occurrence data were aggregated from three principal sources: (1) the Chinese Virtual Herbarium (CVH; https://www.cvh.ac.cn), providing digitized botanical specimen records (accessed 10 Sep 2024); (2) the Global Biodiversity Information Facility (GBIF; https://www.gbif.org), a transnational repository consolidating biodiversity observations (accessed 12 Sep 2024); and (3) China's National Specimen Information Infrastructure (NSII; www.nsii.org.cn), archiving standardized specimen metadata (accessed 15 Sep 2024), which provides extensive biological specimen information. Additional occurrence records were acquired from four multidisciplinary academic platforms: China National Knowledge Infrastructure (CNKI; https://www.cnki.net), Wanfang Data (https://www.wanfangdata.com.cn), VIP Information (https://www.cqvip.com), and Web of Science (WoS; https://www.webofscience.com), with data retrieval conducted from September 18 to 21, 2024. Through meticulous integration and analysis of the information in these databases, a total of 691 non‐redundant distribution records of *L. christinae* in China were collected and confirmed.

**FIGURE 1:**
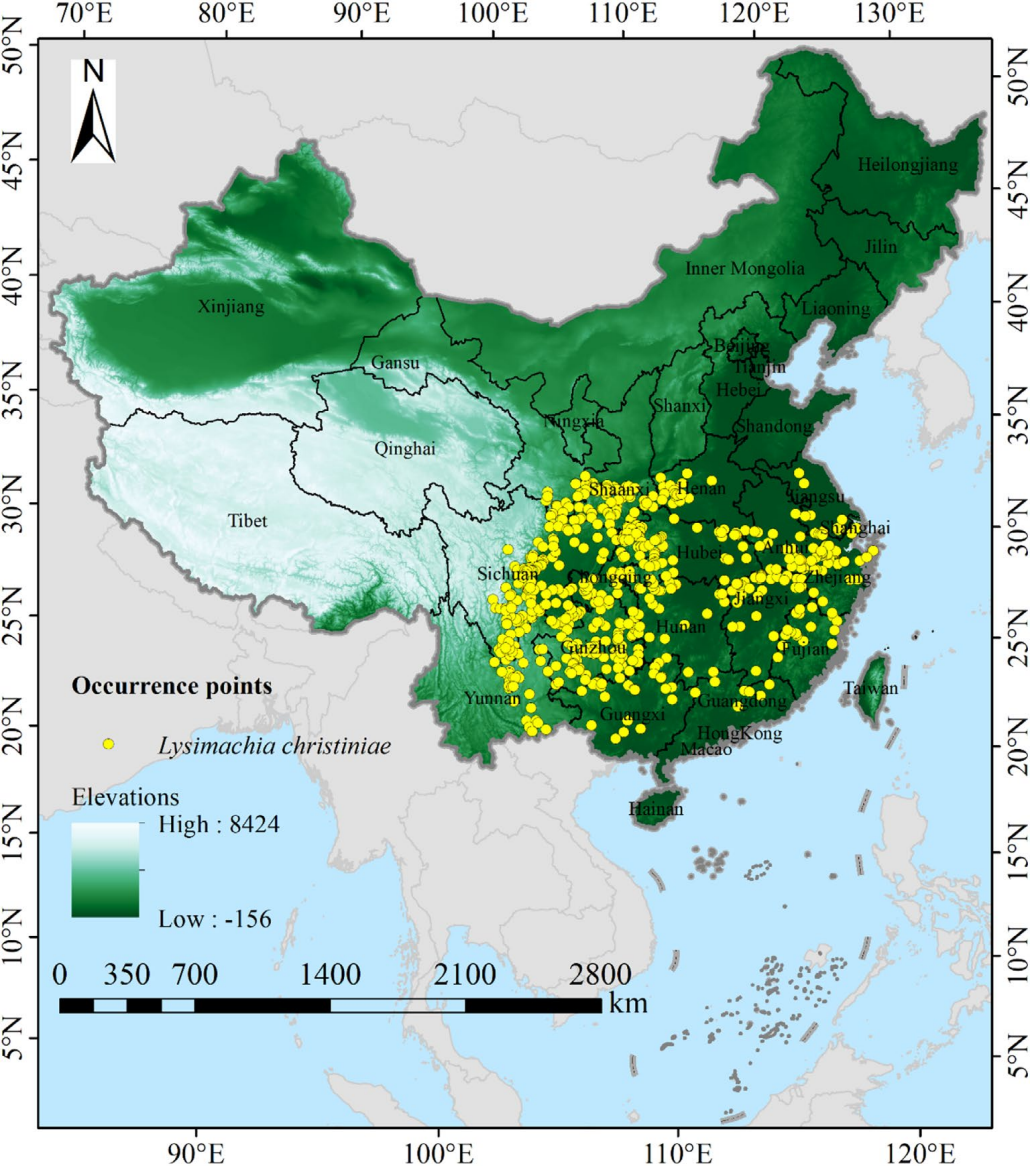
Geographic distribution of 625 records of *L. christinae* in China.

To mitigate the risk of model overfitting and to eliminate redundant distribution data, a grid file with a resolution of 2.5′ × 2.5′ was created in ArcGIS 10.8 software for our research, and the occurrence points of *L. christinae* were subjected to meticulous manual selection. Through geospatial processing algorithms embedded in the software, the minimum Euclidean distance between individual occurrence coordinates and their respective grid centroids was rigorously computed, thereby enforcing a single maximally representative sampling node per grid unit to mitigate spatial autocorrelation. After implementing a spatially explicit filtering protocol, 625 non‐redundant occurrence coordinates were finalized (Figure 1). For compatibility with MaxEnt v3.4.1's input specifications (software portal: biodiversityinformatics.amnh.org, retrieved on 25‐Sep‐2024), the geographical distribution point data file of *L. christinae* was converted from .xls to .csv format.

### 2.2 Compiling and Screening of Climatic Data

Climatic predictors for this analysis comprised 19 bioclimatic indices (Bio1–Bio19), representing temperature and precipitation extremes, sourced from WorldClim v2.1 (https://www.worldclim.org/data/worldclim21.html, retrieved on October 8, 2024), which offer current and future climate scenarios. These factors are all spatially resolved at a 2.5′ × 2.5′ resolution, with the original files in GeoTiff (.tif) format. The current climate data is based on the version 2.0 dataset spanning from 1970 to 2000. Future habitat projections integrated 20‐year climatological means spanning 2041–2100, partitioned into three intervals (2050s: 2041–2060; 2070s: 2061–2080; 2090s: 2081–2100). Climate layers were extracted from the Beijing Climate Center Climate System Model (BCC‐CSM2‐MR) under CMIP6 protocols, which provides globally harmonized atmospheric projections for ecological niche modeling, selected for its applicability to Chinese climate change studies (Tan et al. 2022). Three representative Shared Socioeconomic Pathways (SSPs) under the CMIP6 framework were selected: (1) SSP126, characterized by resilient socioeconomic frameworks and minimal mitigation challenges, achieving a radiative forcing of 2.6 W·m^−2^ by 2100 through rapid decarbonization; (2) SSP370, fragmented governance systems with moderate emission trajectories, culminating in 7.0 W·m^−2^ radiative forcing by century's end; and (3) SSP585 Reflects energy‐intensive growth patterns, projecting 8.5 W·m^−2^ radiative forcing through unconstrained carbon emissions (He, Ma, and Chen 2023). These SSP‐driven projections deliver multivariate bioclimatic covariates across divergent emission trajectories, enabling the simulation of climate‐driven alterations in species' biogeographical ranges under anthropogenic forcing regimes. To ensure compatibility with subsequent analytical processes, these climatic parameters were uniformly transformed into .asc format via ArcGIS 10.8 software in this study.

**FIGURE 2:**
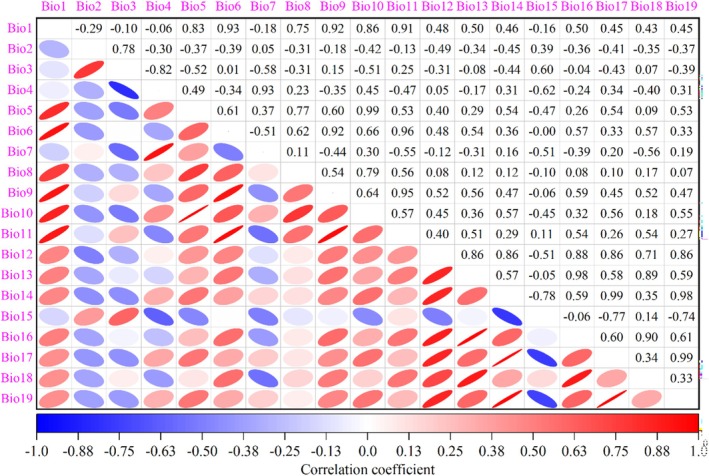
Correlation analysis of the 19 bioclimatic variables of *L. christinae* . Shorter minor axes of ellipses are correlated with larger absolute values of correlation coefficients between factors. Deeper blue saturation indicates stronger negative correlations, while deeper red saturation suggests stronger positive correlations.

Utilizing the “Extract Multi Values to Points” functionality within ArcGIS 10.8 software, the environmental variable data corresponding to the geographical information of 625 distribution points were obtained and subsequently imported into IBM SPSS Statistics 26 software for Pearson correlation analysis (Figure 2). In this study, environmental factors with correlation values less than |0.75| were initially excluded to lessen the effects of multicollinearity. Environmental predictors exhibiting pairwise correlations exceeding |0.75| were subjected to a variance inflation analysis, wherein the explanatory capacity rankings of these 19 bioclimatic covariates on *L. christinae* habitat suitability (Table S1) served as the primary selection criterion, retaining only variables with maximum ecological relevance. Following this meticulous selection process, six representative climate variables were ultimately identified for use in subsequent predictive analyses, which specifically include: Temperature seasonality standard deviation (Bio4), Coldest month minimum temperature (Bio6), Warmest quarter mean temperature (Bio10), Yearly precipitation (Bio12), Seasonal precipitation variability (Bio15), Warmest quarter precipitation (Bio18).

### 2.3 Optimization of MaxEnt Model

While analyzing how climate change impacts the spatial distribution of species using MaxEnt software, it is frequently observed that models are prone to overfitting and exhibit limited transferability (Lissovsky and Dudov 2021; Radosavljevic and Anderson 2014). To address these challenges, the MaxEnt model's feature combinations (FC) and regularization multipliers (RM) have been meticulously optimized in this study through the ENMeval package within R software (Kass et al. 2021; Muscarella et al. 2014). The MaxEnt algorithm was parameterized with five foundational feature classes: linear (L), quadratic (Q), hinge (H), product (P), and threshold (T) functions. Through combinatorial optimization, nine distinct feature sets were generated to balance model complexity, ranging from minimal (e.g., H‐only) to multi‐feature configurations (e.g., LQHPT). Additionally, a geometrically spaced RM sequence (0.5, 1.0, 1.5, …, 4.0) was evaluated to calibrate model hyperparameters. The delta‐corrected Akaike Information Criterion (ΔAICc) was employed to quantify the trade‐off between predictive accuracy and overfitting risks across candidate configurations, following ENMeval's model selection protocol (Kass et al. 2021). Through rigorous selection of the optimal model (minimum delta‐corrected Akaike Information Criterion), overfitting risks were mitigated while predictive performance was optimized. This calibration strategy enhanced the MaxEnt framework's generalization capacity and spatiotemporal transferability across heterogeneous environmental scenarios.

### 2.4 Model Implementation and Validation

To delineate climatically viable habitats for *L. christinae* , we implemented a calibrated MaxEnt species distribution model (SDM) integrating environmental covariates, generating spatially explicit projections of biogeographical suitability under current bioclimatic regimes. To guarantee the precision and dependability of the model, a stochastic sampling approach was employed in this study to partition the sample occurrence points of *L. christinae* into training and validation subsets, with 75% of the data utilized for model calibration and 25% reserved as an independent dataset to assess the model's effectiveness. While developing the model, this study meticulously selected refined RM and FC settings, and the model was executed tenfold to confirm the consistency of the outcomes. Additionally, a logistic regression model was chosen as the model output in this study to facilitate the intuitive display of predicted results.

Model validation employed the area under the receiver operating characteristic curve (AUC‐ROC), a threshold‐independent metric widely endorsed in ecological niche modeling. The AUC‐ROC quantifies a model's discriminatory power by measuring its capacity to differentiate species presence localities from environmentally stratified pseudo‐absences, with values > 0.9 indicating high predictive reliability. This metric's robustness stems from its invariance to prevalence imbalances in presence‐background data, effectively capturing species‐environment associations independent of sampling biases. The AUC‐ROC score serves as a robust proxy for model efficacy, where elevated values signify enhanced discriminatory capacity in resolving species‐environment relationships. Following the classification framework for diagnostic tests of Dong et al. (2025), predictive performance was stratified into five tiers: exceptional (0.9–1.0), strong (0.8–0.9), moderate (0.7–0.8), marginal (0.6–0.7), and unreliable (0.5–0.6), with higher scores reflecting improved concordance between predicted and observed biogeographical patterns.

### 2.5 Classification of the Suitable Habitat for *L. christinae*

To classify the suitable habitat for *L. christinae* , we employed the “Maximum test sensitivity plus specificity” threshold option in the MaxEnt model (Elith et al. 2011; Phillips et al. 2006). This threshold method is widely used for its balance between sensitivity and specificity, ensuring that the model effectively distinguishes between suitable and unsuitable habitats while minimizing false positives and false negatives (Chen et al. 2025; Yang, Zhu, et al. 2024). This approach was deemed appropriate for our study as it provides a robust threshold for predicting the species' potential distribution under various climatic scenarios. Spatial suitability thresholds were derived from decadal cross‐validated outputs of the MaxEnt ensemble model (10 replicates, 2.5′ × 2.5′ resolution), with aggregated probability surfaces (0–1 scale) informing habitat classification under a spatially explicit framework (Elith et al. 2011). The data conversion capabilities of ArcGIS 10.8 software were utilized to transform the MaxEnt model's output data from .asc format to .tif format. The Natural Breaks classification method was adopted to delineate the likely conducive environments for *L. christinae* , stratifying habitat suitability into four hierarchical tiers based on spatially explicit ecological thresholds: non‐suitable areas (*p* < 0.1), generally suitable areas (0.1 ≤ *p* < 0.3), moderately suitable areas (0.3 ≤ *p* < 0.5), and high suitable areas (*p* ≥ 0.5).

### 2.6 Geospatial Dynamics in *L. christinae* Habitat Suitability

To project range dynamics of *L. christinae* under CMIP6 climate trajectories (2041–2100), we developed a multi‐temporal ensemble model that quantified habitat suitability transitions across three SSP scenarios. Spatiotemporal shifts were classified as range expansions (newly suitable zones), contractions (habitat losses), and stable refugia (persistent suitability), employing a spatially explicit framework to disentangle climate‐driven redistribution patterns (Thuiller et al. 2005). Based on the suitability threshold of ≥ 0.1 (Zhou et al. 2021), a binary matrix of “presence/absence” (1/0) was established, and the transformation of matrix elements (0 → 1, 1 → 0, 1 → 1) was used to visually reflect the patterns of change in suitable habitats: (1) Expansion areas (0 → 1) indicate regions that were not originally suitable for *L. christinae* to grow but are anticipated to be favorable in the future, suggesting potential areas for expansion of growth. (2) Contraction areas (1 → 0) reveal regions that, under the influence of climate change, are gradually becoming unsuitable for *L. christinae* , highlighting the urgency of ecological conservation. (3) Stable areas (1 → 1) represent regions that maintain their suitability over different time periods, which are crucial for the stable persistence of *L. christinae* populations.

### 2.7 Centroid Migration of *L. christinae*

Following established methods for tracking species range shifts under climate change (Liu et al. 2025; Yang, Xiang, et al. 2024), the spatial‐statistical assessment of bioclimatic niche centroid shifts for *L. christinae* was conducted through a five‐stage GIS‐based workflow: (1) The species distribution probability files predicted by the MaxEnt model, covering the present and future three temporal intervals across various climatic conditions, are loaded into ArcGIS 10.8 for processing. (2) A threshold of presence probability not less than 0.1 for *L. christinae* is used to classify geographical units into suitable and unsuitable living areas through the “Reclassify (Spatial Analyst)” tool, and the suitable distribution area layer files are exported. (3) Based on the exported suitable distribution area layer files, the “raster to point” tool is first used to convert the suitable distribution areas into point data, followed by the application of the “mean center” tool to derive the geometric center coordinates of each appropriate distribution zone. (4) The “points to line” tool is utilized to plot the trajectory of the geometric center of *L. christinae* 's suitable living areas across time, based on the calculated centroid coordinates. (5) The “point distance” tool is employed to measure the distances between centroids at different time periods, quantifying the spatial changes in centroid migration.

## 3 Results

### 3.1 Model Calibration and Predictive Validation

**FIGURE 3:**
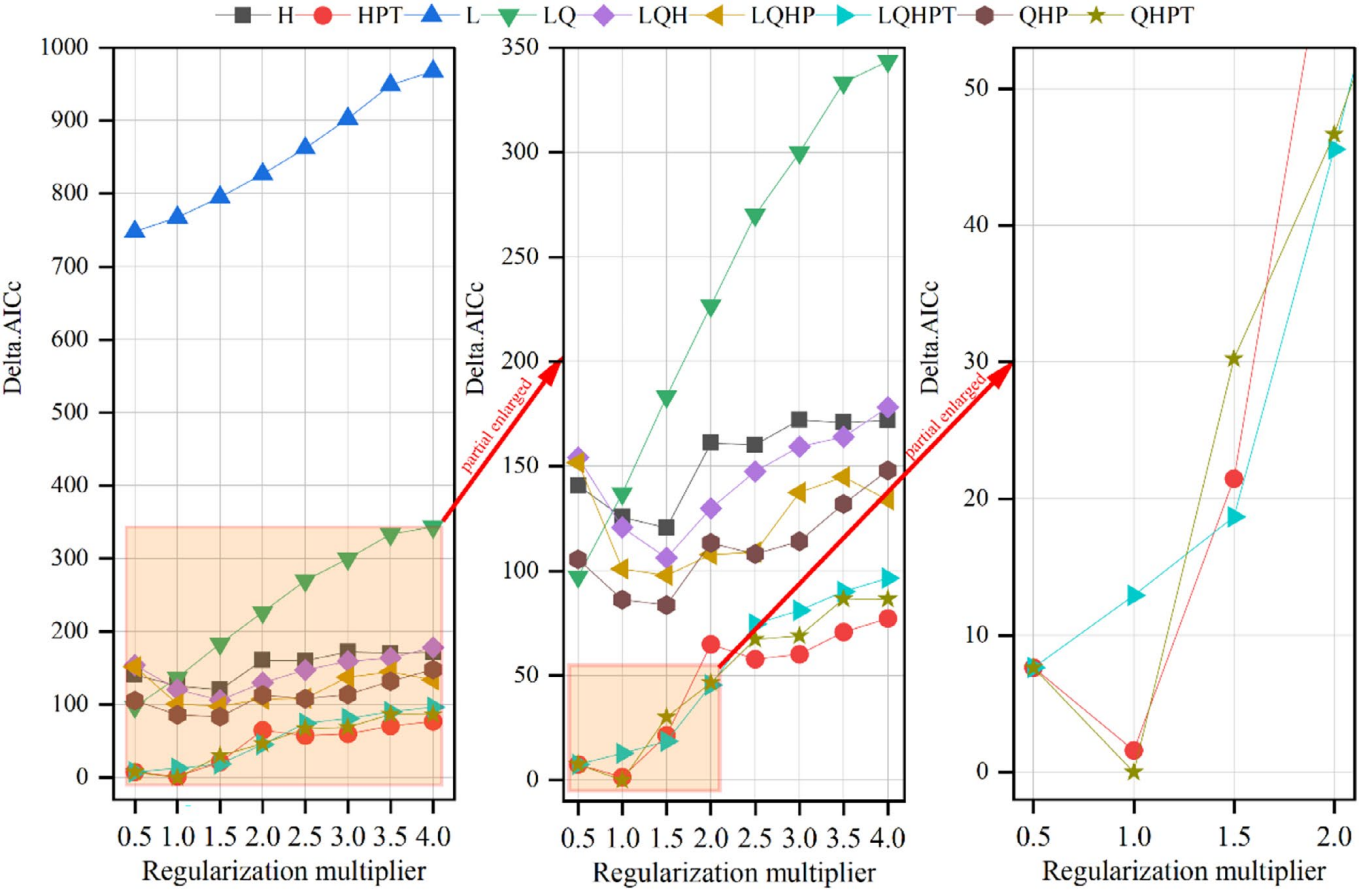
ENMeval‐derived Delta.AICc for *L. christinae* . The symbols denote different feature categories (*L: Linear features, Q: Quadratic features, H: Hinge features, P: Product features, T: Threshold features*).

**FIGURE 4:**
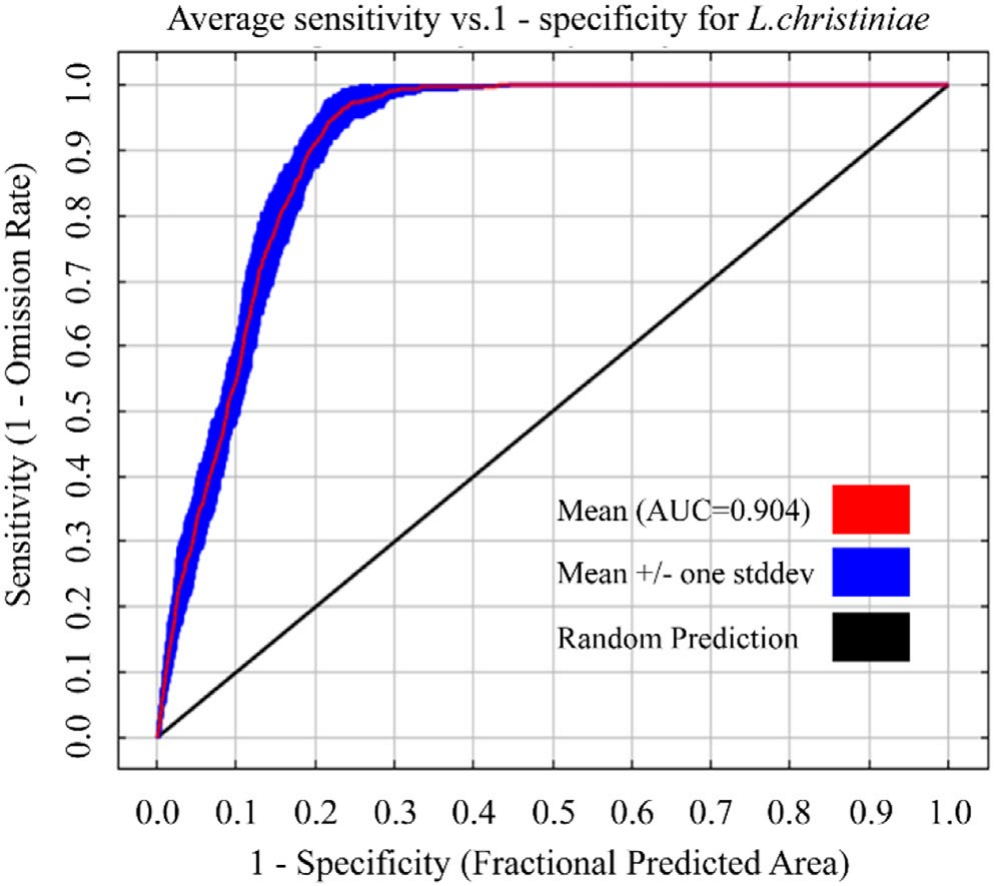
AUC‐ROC curve of MaxEnt‐based habitat suitability model for *L. christinae* .

Based on 625 georeferenced occurrence records and six bioclimatic covariates, we implemented a MaxEnt 3.4.1 workflow to project *L. christinae* 's potential distribution. Initial runs with default parameters (RM = 1, FC = LQHPT, delta. AICc = 12.95) were compared against optimized configurations using the ENMeval package. The parameter set minimizing overfitting (RM = 1.0, FC = QHPT, delta.AICc = 0) demonstrated superior predictive accuracy through AICc‐based model selection (Figure 3). Species distribution projections were generated through MaxEnt v3.4.1, employing ENMeval‐optimized hyperparameters to balance model complexity: regularization strength (RM = 1.0) and feature space configuration (FC = QHPT). To assess model stability, we conducted 10 independent cross‐validation runs, yielding a mean AUC‐ROC score of 0.904 (95% CI: 0.890–0.924) (Figure 4). Based on AUC evaluation standards, this value signifies an exceptionally high level of predictive precision.

### 3.2 Dominant Environmental Variable

**FIGURE 5:**
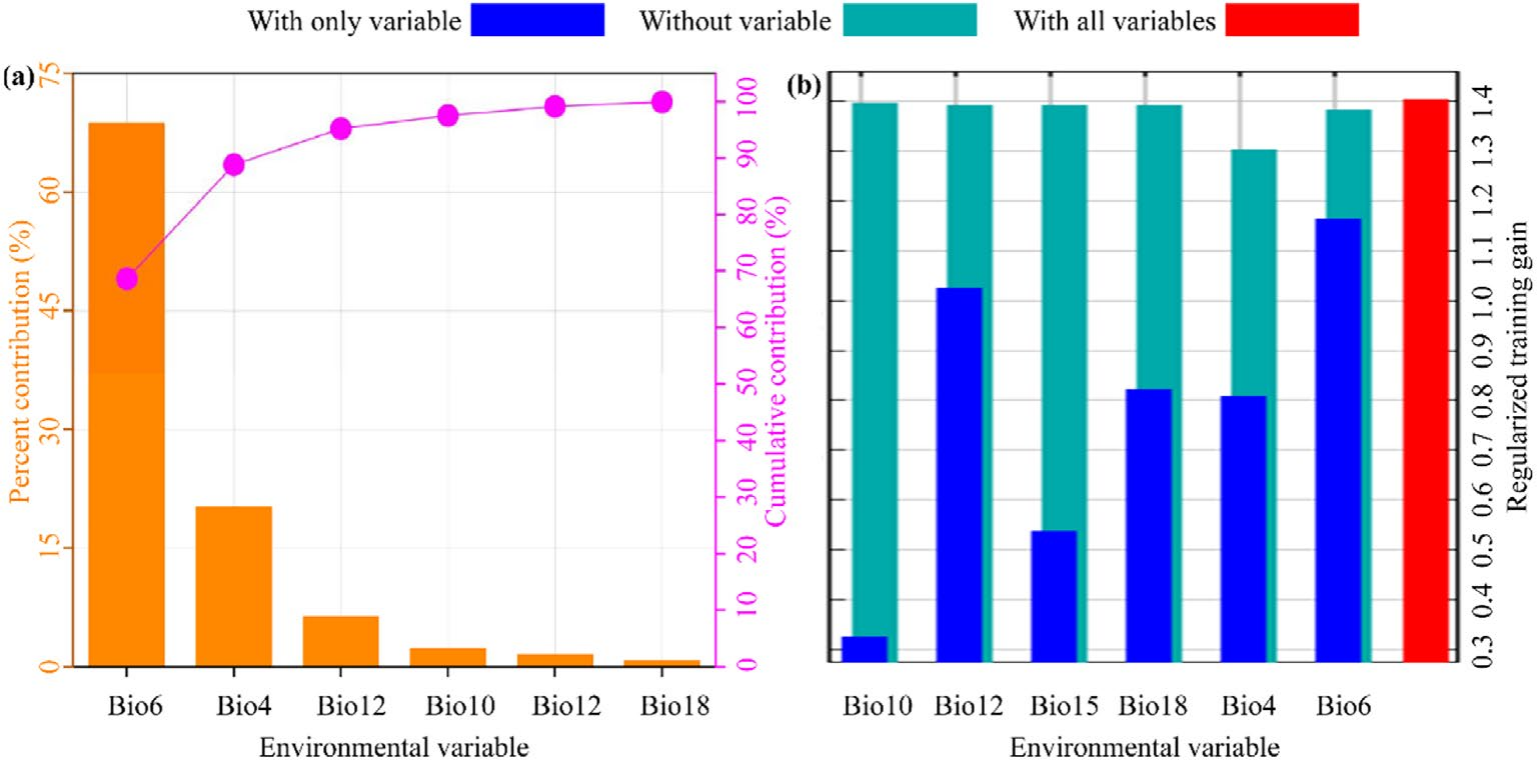
MaxEnt‐derived metrics for *L. christinae* habitat determinants: (a) relative contribution weights of bioclimatic variables; (b) regularization parameter optimization via Jackknife‐based training gain diagnostics.

To identify dominant environmental drivers shaping the biogeographical patterns of *L. christinae* , we implemented a MaxEnt 3.4.1 framework with a dual‐validation approach: (1) Variable importance quantification via permutation‐based contribution analysis; and (2) Feature selection optimization using jackknife resampling of regularized training gains. Through the analysis of the MaxEnt model, six key environmental factors influencing the distribution of *L. christinae* were identified (Figure 5a), with the following contribution rates: the min temperature of coldest month (Bio6) contributes 68.7%, making it the most significant influencing factor; the standard deviation of temperature seasonality (Bio4) contributes 20.2%, as the second most influential factor; annual precipitation (Bio12) contributes 6.4%; the average temperature of the warmest month (Bio10) contributes 2.3%; the seasonality of precipitation (Bio15) contributes 1.6%; and the precipitation of the warmest month (Bio18) contributes 0.8%. In the jackknife test (Figure 5b), the min temperature of the coldest month (Bio6) exhibited the highest regularized training gain when evaluated individually, indicating its status as the most informative variable. Conversely, the removal of the standard deviation of temperature seasonality (Bio4) resulted in the most substantial reduction in regularized training gain, implying that it holds unique information not captured by other variables. In accordance with the criterion that the cumulative contribution rate exceeds 85% (Huang et al. 2024), Bio6 and Bio4 were determined to be the primary ecological drivers, with their combined model contribution rate reaching 88.9%, significantly influencing the geographical distribution of *L. christinae* in China.

**FIGURE 6:**
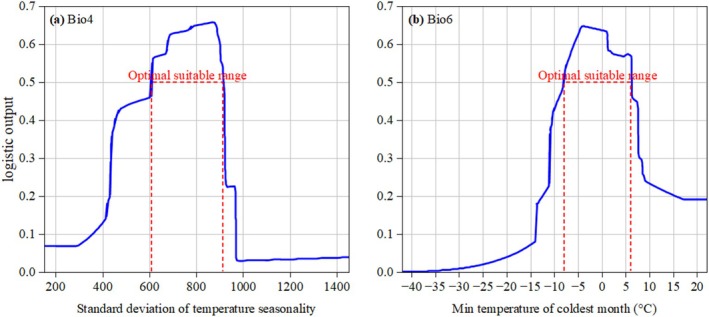
Marginal effect curves of dominant predictors on *L. christinae* spatial suitability.

According to Figure 6a, the distribution probability of *L. christinae* increases with the increase in standard deviation of temperature seasonality within the range of 295.83 to 876.21. Beyond 876.21, the distribution probability of *L. christinae* decreases with the further increase in standard deviation of temperature seasonality, reaching its minimum value of 0.03 when the standard deviation of temperature seasonality reaches 983.27. In addition, Figure 6b demonstrates that the distribution probability of *L. christinae* approaches zero when the min temperature of the coldest month falls below −35°C. Within the range of −35°C to −3.91°C, there is a positive correlation between the min temperature of the coldest month and the distribution probability, which increases as the temperature rises. Conversely, beyond −3.91°C, the distribution probability exhibits a negative correlation with the min temperature of the coldest month, declining as the temperature continues to increase. The interplay between environmental factors and the likelihood of species inhabiting suitable habitats is illuminated by response curves, where regions with a distribution probability above 0.5 are generally considered favorable for species growth (Wang et al. 2023). Based on this, it has been found that the optimal growth conditions for *L. christinae* occur when the standard deviation of temperature seasonality is between 607.62 and 915.65, and the min temperature of the coldest month ranged from −8.12°C to 6.21°C.

### 3.3 Bioclimatic Suitability Mapping for *L. christinae* Under Baseline Climate Conditions

**FIGURE 7:**
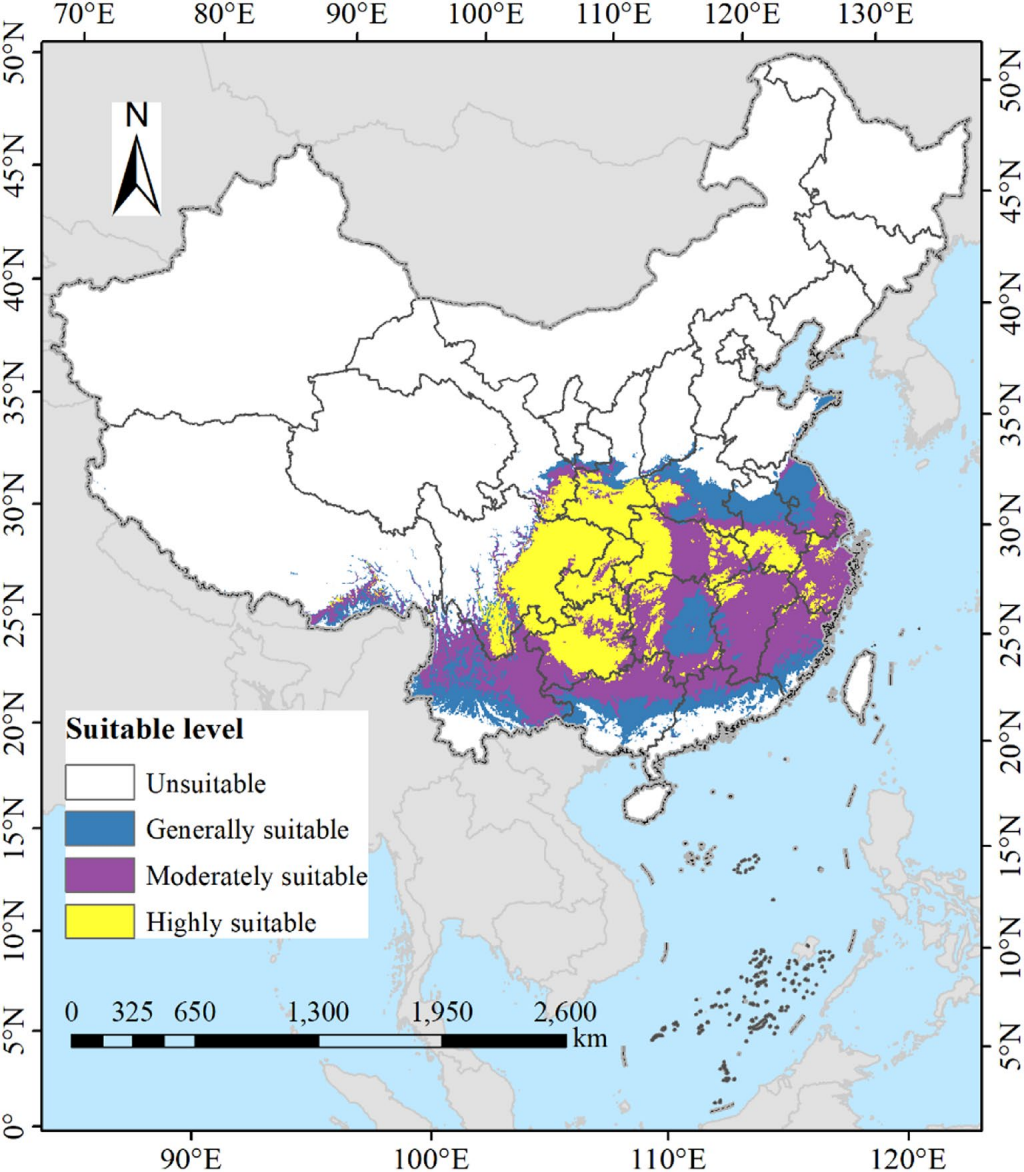
Geospatial projections of *L. christinae* habitat range in China constrained by present‐day climatic regimes.

Under current climatic conditions (Figure 7), the total potential suitable habitat area for *L. christinae* is calculated to be 223.90 × 10^4^ km^2^, accounting for 23.32% of China's total land area. Specifically, the area of highly suitable habitats is approximately 72.12 × 10^4^ km^2^, constituting 7.51% of the national land area, and is predominantly found in regions such as Chongqing Municipality, Guizhou Province, eastern Sichuan Province, southern Gansu Province, southern Shaanxi Province, western Hubei Province, northwestern Hunan Province, and southern Anhui Province, among others. The area of moderately suitable habitats spans about 97.44 × 10^4^ km^2^, representing 10.15% of the land area, and includes most parts of Jiangxi Province and Zhejiang Province, northern Yunnan Province, southwestern Guizhou Province, northern Guangxi Zhuang Autonomous Region, northern Guangdong Province, southern and northwestern Hunan Province, northern Fujian Province, central Hubei Province, and southeastern Jiangsu Province. While the area of generally suitable habitats reaches 54.34 × 10^4^ km^2^, making up 5.66% of the land area, and is mainly distributed in central Yunnan Province, central Guangxi Zhuang Autonomous Region, central Guangdong Province, central Hunan Province, central Fujian Province, central Anhui Province, northern Jiangsu Province, and southern Henan Province. As for the non‐suitable areas, they are primarily distributed across the vast regions of Northwest and North China, Taiwan Province, Hainan Province, as well as the extensive areas south of Yunnan Province, Guangxi Zhuang Autonomous Region, Guangdong Province, and Fujian Province. The MaxEnt model predictions align well with the known distribution of *L. christinae* , with a concordance rate of approximately 92% between predicted highly suitable areas and documented occurrence records. Minor discrepancies occur in peripheral areas where the species may be under sampled or where microhabitat conditions not captured by our 2.5′ resolution climate data may influence actual presence.

### 3.4 Projected Geographic Spread of *L. christinae* in China Across Diverse Climatic Projections

**FIGURE 8:**
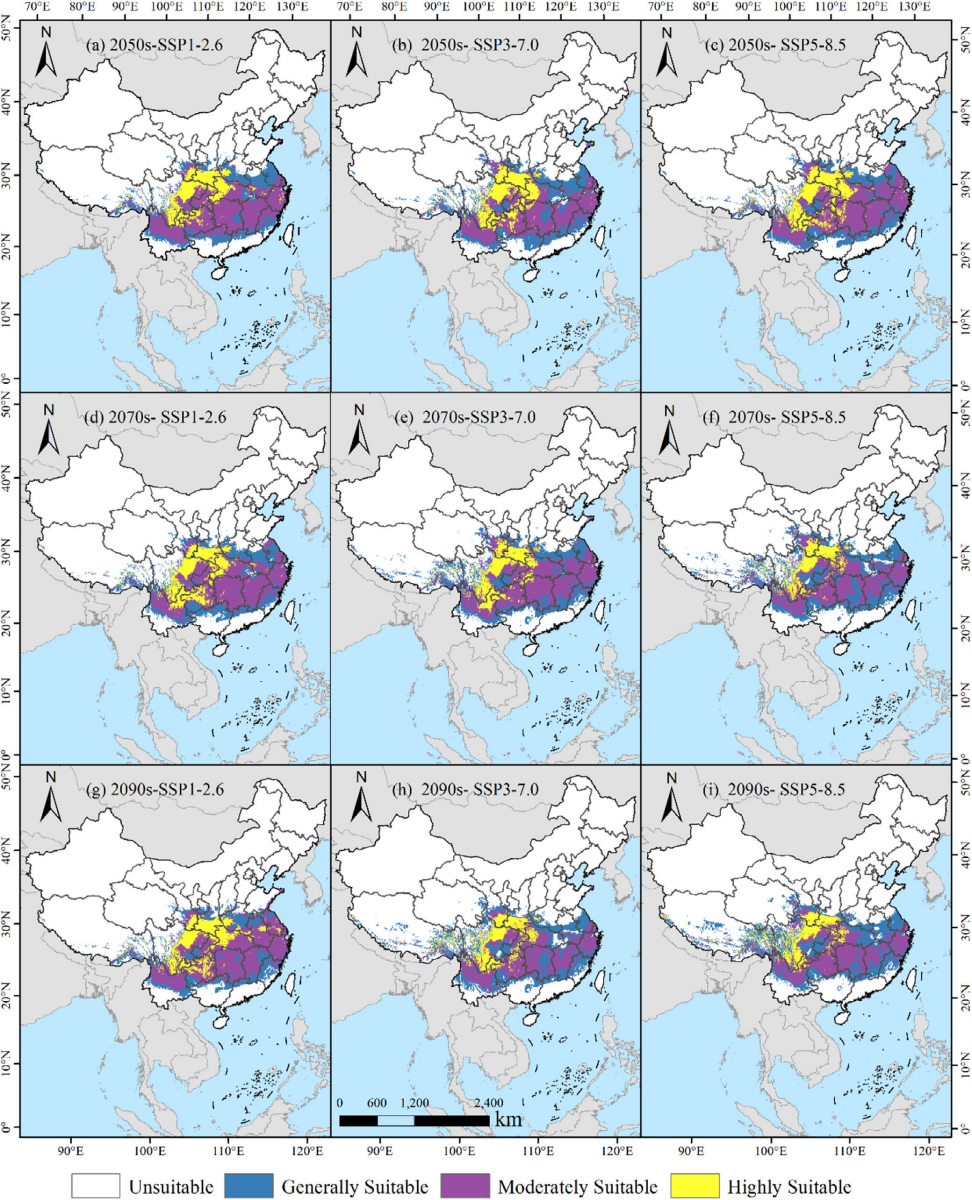
Projected geographic spread of *L. christinae* in China across diverse climatic projections.

In the SSP1‐2.6 climate scenario, the total potential suitable habitats for *L. christinae* in China during the 2050s are calculated to be 216.22 × 10^4^ km^2^, accounting for 22.52% of China's total land area (Table 1). The suitable habitats are divided into low, medium, and high levels, with areas of 58.40 × 10^4^ km^2^, 114.80 × 10^4^ km^2^, and 43.02 × 10^4^ km^2^, respectively. Highly suitable areas are predominantly located in the eastern Sichuan Province, the northwestern Guizhou Province, the northeastern Yunnan Province, the western Hubei Province, the southern Gansu Province, the western Henan Province, and the southern Shaanxi Province (Figure 8a,d,g). These regions, due to their unique geographical and climatic conditions, provide a highly favorable environment for the growth of *L. christinae* . Further analysis indicates that in the 2070s and 2090s, both the total suitable habitats and the high suitable area of *L. christinae* show trends of change, with the area of high suitable areas increasing year by year, suggesting that the environmental conditions in these regions are becoming more favorable for the suitability of *L. christinae* .

**TABLE 1:** Projected suitable habitat range of *L. christinae* across various climatic projections. (units: 10^4^ km^2^).

Period	Total suitable area	Generally suitable area	Moderately suitable area	Highly suitable area
Current	223.90	54.34	97.44	72.12
SSP1‐2.6‐2050s	216.22	58.40	114.80	43.02
SSP1‐2.6‐2070s	214.12	60.87	107.70	45.55
SSP1‐2.6‐2090s	223.80	60.09	116.87	46.85
SSP3‐7.0‐2050s	218.65	74.18	97.67	46.80
SSP3‐7.0‐2070s	226.27	77.59	108.84	39.83
SSP3‐7.0‐2090s	222.80	91.55	93.53	37.72
SSP5‐8.5‐2050s	223.19	66.06	109.19	47.94
SSP5‐8.5‐2070s	216.25	90.41	94.02	31.82
SSP5‐8.5‐2090s	226.89	98.42	92.88	35.58

Under the SSP3‐7.0 climate scenario, the total suitable habitats for *L. christinae* in the 2050s is 218.65 × 10^4^ km^2^, representing 22.78% of China's land area (Table 1). Compared with the SSP1‐2.6 scenario, the areas of low, medium, and high suitability are 74.18 × 10^4^ km^2^, 97.67 × 10^4^ km^2^, and 46.80 × 10^4^ km^2^, respectively. The distribution of highly suitable areas has changed, with the addition of areas such as the northeastern Chongqing. However, as time progresses to the 2070s and 2090s, while the total suitable habitats remain relatively stable, the area of high suitable areas shows a decreasing trend, and the area of generally suitable areas increases year by year. This may indicate that the originally most favorable environmental conditions are undergoing changes, posing potential threats to the survival of *L. christinae* . Specifically, in the 2070s, high suitable areas are mainly distributed in the eastern Sichuan Province, the western Guizhou Province, the northeastern Chongqing, the northeastern Yunnan Province, the western Hubei Province, the western Henan Province, the southern Gansu Province, the southern Shaanxi Province, and a small area in the northwestern Hunan Province; by the 2090s, high suitable areas are further reduced to the eastern Sichuan Province, the northeastern Chongqing, the northeastern Yunnan Province, the western Hubei Province, the western Henan Province, the southern Gansu Province, and the southern Shaanxi Province (Figure 8b,e,h).

In the SSP5‐8.5 climate scenario, the total suitable habitats for *L. christinae* in the 2050s are 223.19 × 10^4^ km^2^, constituting 23.25% of China's total land area, with low, medium, and high suitable areas of 66.06 × 10^4^ km^2^, 109.19 × 10^4^ km^2^, and 47.94 × 10^4^ km^2^, respectively (Table 1). Specifically, in the 2050s, high suitable areas are mainly distributed in the eastern Sichuan Province, the central Guizhou Province, northeastern Chongqing, western Hunan Province, northeastern Yunnan Province, western Hubei Province, western Henan Province, southern Gansu Province, and southern Shaanxi Province; in the 2070s, high suitable areas are mainly distributed in the eastern Sichuan Province, western Guizhou Province, northeastern Chongqing, northeastern Yunnan Province, western Hubei Province, western Henan Province, southern Gansu Province, and southern Shaanxi Province; by the 2090s, high suitable areas are further reduced to the eastern Sichuan Province, northeastern Chongqing, northeastern Yunnan Province, western Hubei Province, southern Gansu Province, and southern Shaanxi Province (Figure 8c,f,i). This trend indicates that under extreme climate scenarios, the distribution of suitable habitats for *L. christinae* may face greater uncertainty, and further research is needed to clarify its adaptability to climate change.

### 3.5 Shifts in Suitable Habitats for *L. christinae* Across Diverse Climatic Projections in China

**FIGURE 9:**
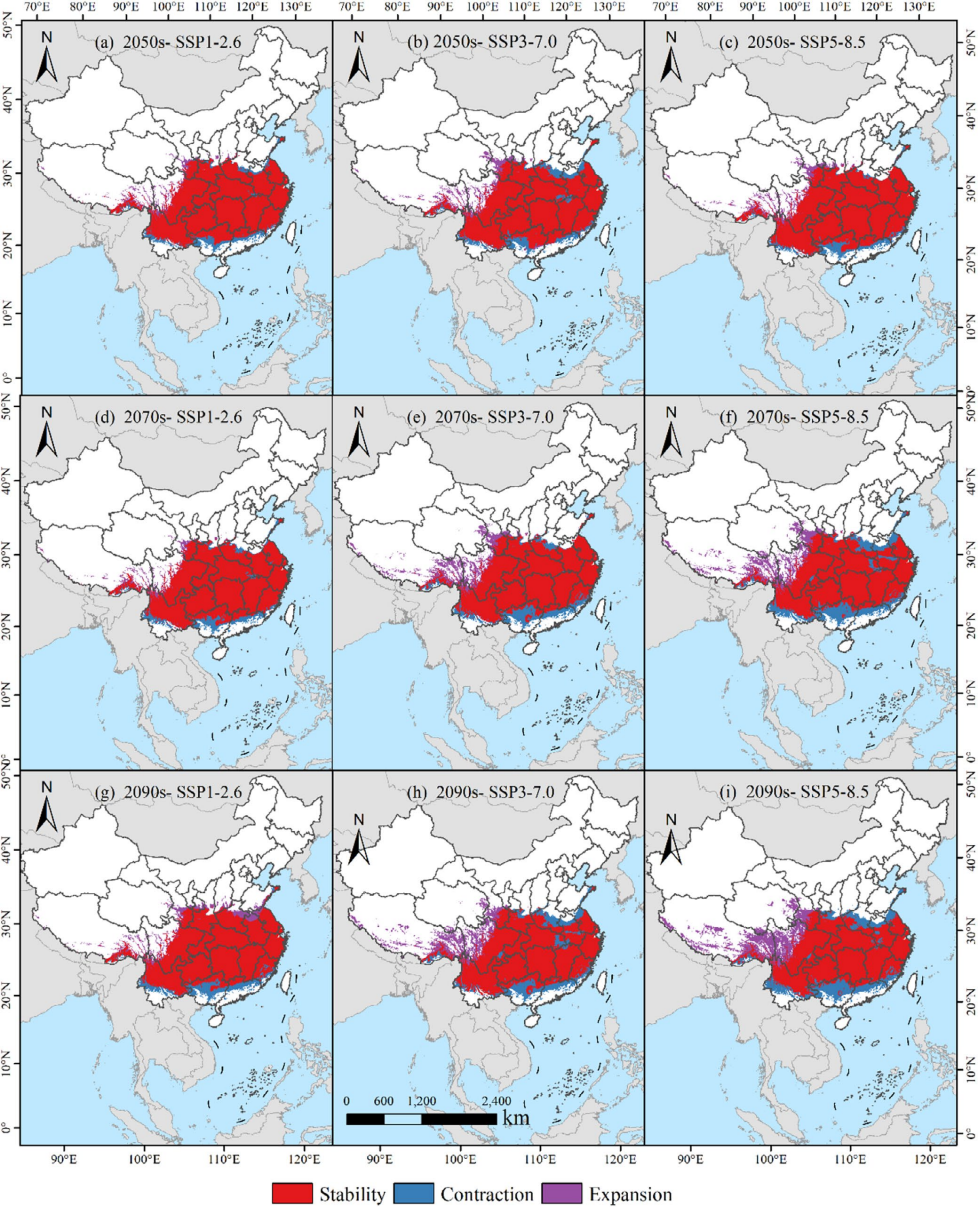
Shifts in the projected geographic spread of *L. christinae* across distinct time periods and climatic conditions.

Under the SSP1‐2.6 climate scenario, the suitable habitat area for *L. christinae* demonstrated a relatively high retention rate across the decades of the 2050s, 2070s, and 2090s, with figures of 86.78%, 86.13%, and 82.03%, respectively (Figure 9a,d,g; Table 2). These data indicate that, under this low‐emission scenario, the suitable habitat for *L. christinae* remained relatively stable, with low loss rates of 8.22%, 9.01%, and 9.02%. Concurrently, the area of suitable habitat increase showed a gradual decline over the three periods, with 14.55 × 10^4^ km^2^, 14.12 × 10^4^ km^2^, and 27.18 × 10^4^ km^2^, and the increase rates dropped from 5% to 4.86%, then rose to 8.95%. This may reflect that, under a lower greenhouse gas emission scenario, the distribution of *L. christinae* was less affected and showed a certain expansion trend.

**TABLE 2:** Dynamics of shifts in the habitable zones of *L. christinae* across distinct temporal phases and climatic conditions.

Period	Area (10^4^ km^2^)			Rate of change (%)		
	Stability	Contraction	Expansion	Stability	Contraction	Expansion
SSP1‐2.6‐2050s	252.49	23.91	14.55	86.78	8.22	5.00
SSP1‐2.6‐2070s	250.22	26.18	14.12	86.13	9.01	4.86
SSP1‐2.6‐2090s	249.03	27.39	27.18	82.03	9.02	8.95
SSP3‐7.0‐2050s	245.26	31.14	24.79	81.43	10.34	8.23
SSP3‐7.0‐2070s	241.84	34.57	37.67	77.00	11.01	11.99
SSP3‐7.0‐2090s	230.44	45.93	44.83	71.74	14.30	13.96
SSP5‐8.5‐2050s	252.73	23.66	22.91	84.44	7.910	7.65
SSP5‐8.5‐2070s	222.96	53.45	44.06	69.57	16.68	13.75
SSP5‐8.5‐2090s	218.82	57.58	61.46	64.77	17.04	18.19

Under the SSP3‐7.0 climate scenario, the retention rate of the suitable habitat for *L. christinae* decreased from 81.43% in the 2050s to 71.74% in the 2090s (Table 2; Figure 9b,e,h), exhibiting a decreasing trend over time. Simultaneously, the loss rate increased from 10.34% to 14.30%, indicating that under this medium‐emission scenario, the suitable habitat for *L. christinae* was increasingly negatively impacted. The area of suitable habitat increase was 24.79 × 10^4^ km^2^ in the 2050s, with an increase rate of 8.23%, and rose to 44.83 × 10^4^ km^2^ in the 2090s, with an increase rate of 13.96%, demonstrating an accelerated trend in the expansion of suitable habitats, which may be a response to climate change leading to alterations in the species' distribution range.

Under the SSP5‐8.5 climate scenario (Figure 9c,f,i), the retention rate of the suitable habitat for *L. christinae* significantly declined from 84.44% in the 2050s to 64.77% in the 2090s, with the loss rate increasing from 7.91% to 17.04% (Table 2), marking the most significant change among the three scenarios. The area of suitable habitat increase rose from 22.91 × 10^4^ km^2^ in the 2050s to 61.46 × 10^4^ km^2^ in the 2090s, with the increase rate escalating from 7.65% to 18.19%. Under this high‐emission scenario, the suitable habitat for *L. christinae* suffered the most negative impact, with an increase in loss area and the most pronounced expansion of suitable habitats, suggesting that under more severe climate change conditions, the instability of species distribution increases and the species may need to adapt to a broader range of climatic conditions.

### 3.6 Geometric Centers of Habitable Zones for *L. christinae* Across Temporal Intervals and Climatic Conditions

**FIGURE 10:**
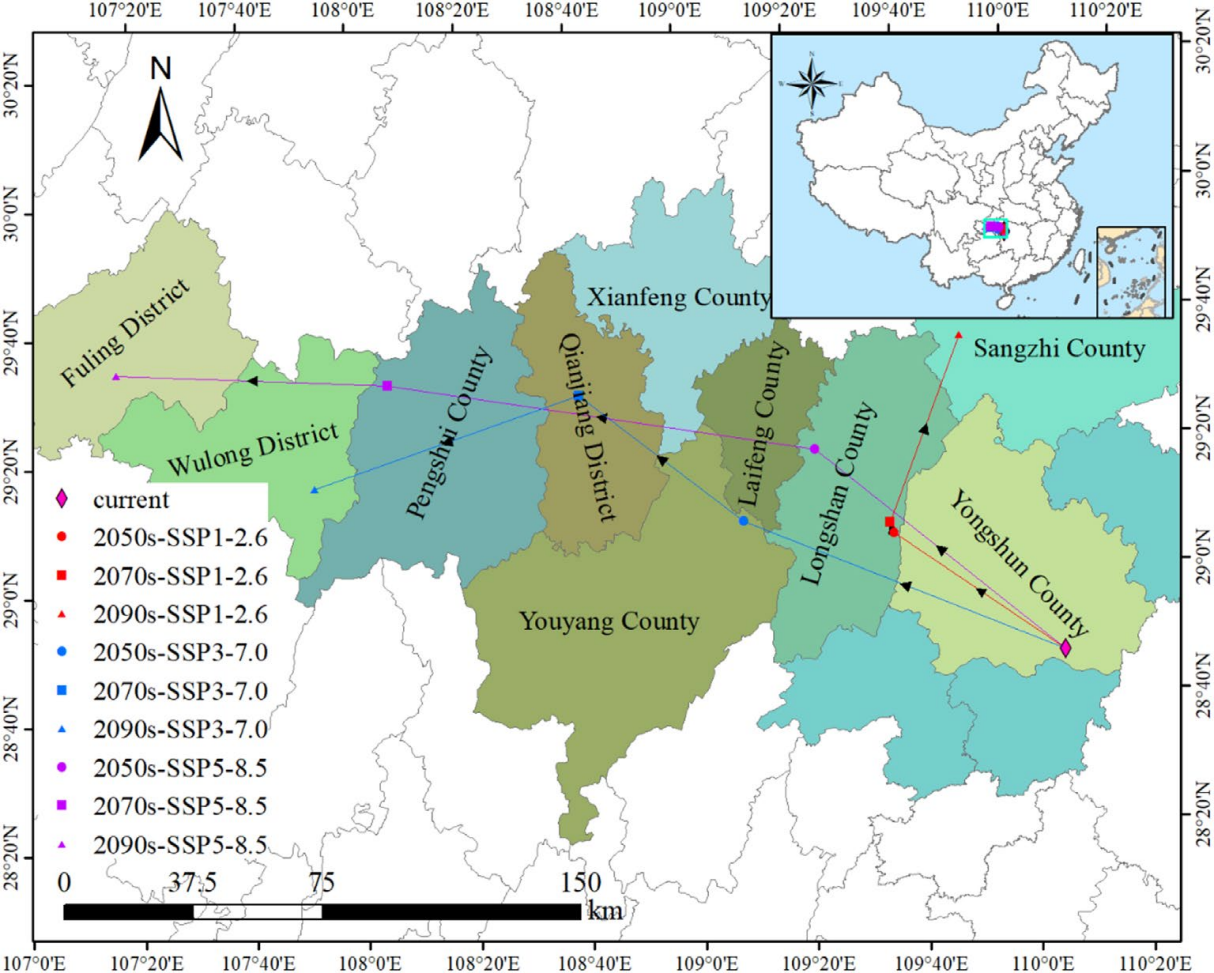
Shifts in the geometric center of *L. christinae* in Hunan Province and Chongqing Municipality across distinct time periods and climatic conditions.

In China, the centroid of the potential appropriate environment for *L. christinae* exhibits a northwestward migration (Figure 10). The present geometric center of the native habitat of *L. christinae* is situated in Xiaoxi Town, Yongshun County, Hunan Province (110°7′ E, 28°47′ N). In the context of the SSP1‐2.6 climate projection, the centroid of the bioclimatic niche for *L. christinae* is expected to move 60.31 km to the northwest from Xiaoxi Town, Yongshun County, to Gaoping Village, Gaoping Town, Longshan County, Hunan Province (109°37′ E, 29°06′ N) during the 2050s. Within the timeframe of the 2070s, an additional northwestward shift of 3.25 km is anticipated, leading to the centroid's relocation to Luota Town, Longshan County (109°36′ E, 29°8′ N). Looking toward the 2090s, the geometric center of the distribution is expected to move 57.69 km to the northeast from Luota Town to Hekou Town, Sangzhi County, Hunan Province (109°50′ E, 29°36′ N). Under the SSP3‐7.0 climate scenario, the distribution centroid of *L. christinae* is expected to shift 101.11 km to the northwest from Xiaoxi Town, Yongshun County to Wufu Town, Youyang County, Chongqing Municipality (109°10′ E, 29°9′ N) during the 2050s. In the 2070s, a northwestward shift of 60.24 km is projected, resulting in the centroid's arrival at Apengjiang Town, Qianjiang District, Chongqing Municipality (108°41′ E, 29°29′ N). By the 2090s, a southwestward movement of 81.96 km is anticipated, with the centroid ultimately locating at Baiyun Town, Wulong District, Chongqing Municipality (107°52′ E, 29°16′ N). Under the SSP5‐8.5 climate scenario, the distribution centroid of *L. christinae* is projected to shift 93.29 km to the northwest from Xiaoxi Town, Yongshun County, to Xiluo Town, Longshan County, Hunan Province (109°23′ E, 29°20′ N) during the 2050s. In the decade of the 2070s, a northwestward shift of 126.28 km is expected, leading to the centroid's relocation to Longshe Town, Pengshui County, Chongqing Municipality (108°6′ E, 29°32′ N). During the 2090s, the distribution centroid is anticipated to move 79.44 km to the northwest from Longshe Town to Mawu Town, Fuling District, Chongqing Municipality (107°17′ E, 29°35′ N).

## 4 Discussion

### 4.1 Refinement and Evaluation of MaxEnt Model for *L. christinae*

Refining model parameters of the MaxEnt framework is essential for improving the predictive precision of species distribution influenced by environmental change (Li et al. 2020; Shi et al. 2024; Xu, Su, and Ren 2024). In this study, the MaxEnt model was optimized through meticulous parameter adjustments, successfully predicting the probable distribution areas of *L. christinae* in China. By modifying the RM and FC, the model achieved ideal performance with an RM set to 1.0 and an FC set to QHPT, resulting in an AUC value of 0.904. This demonstrates a significant degree of predictive precision and robustness. The optimization process effectively mitigated the risk of model overfitting, enhancing its generalization capability under various environmental conditions (Radosavljevic and Anderson 2014; Wen et al. 2024).

Compared to earlier research (Jiang et al. 2023; Ma et al. 2024), this study conducted a more systematic and detailed optimization of model parameters. Many studies rely solely on the default parameter settings of MaxEnt (Shen et al. 2022; Xu et al. 2018), whereas the current study employed the ENMeval package to systematically optimize FC and regularization parameters (Kass et al. 2021; Muscarella et al. 2014), ensuring the model's applicability under various climate scenarios. For instance, Morales et al. (2017) demonstrated that using default MaxEnt settings for *Abies pinsapo* resulted in overpredicted distribution areas with AUC values inflated by 0.12 compared to optimized models. Similarly, Shcheglovitova and Anderson (2013) showed that default parameters led to geographically implausible projections for 60% of their virtual species tests. Conversely, studies implementing systematic optimization have achieved demonstrable improvements: Vignali et al. (2020) reported a 23% reduction in omission error rates through ENMeval optimization for alpine plant species, while Muscarella et al. (2014) documented improved transferability across time periods when using optimized parameters. Furthermore, the stability of the results was verified through multiple model runs, thereby enhancing the reliability of the study. In contrast, some studies may have limited the forecasting reliability and applicability of the model due to a lack of such detailed parameter optimization (Luo et al. 2024; Ma et al. 2024).

### 4.2 Principal Climatic Variables Determining Habitat Appropriateness of *L. christinae*

This study reveals that the min temperature of the coldest month (Bio6) and the standard deviation of temperature seasonality (Bio4) are the principal ecological drivers affecting the distribution of *L. christinae* , with respective contributions of 68.7% and 20.2%. These factors significantly affect the development and spread of *L. christinae* , with the minimum temperature in cold months being crucial for the species' survival. Variations in minimum temperature directly impact the growth cycle and reproductive capacity of *L. christinae* , while the standard deviation of temperature seasonality reflects the magnitude of temperature fluctuations, which can adversely affect the plant's physiological processes (Hedhly 2011; Johnová et al. 2016). Additionally, other climatic factors such as annual precipitation (Bio12) and Variation of precipitation seasonality (Bio15) also exert certain influences on the distribution of *L. christinae* , albeit to a lesser extent. The synergistic interplay of bioclimatic covariates sculpts the spatial distribution patterns of *L. christinae* in China, mechanistically mediating its niche optimization and demographic processes.

Our analytical outcomes decode the niche conservatism and phenotypic plasticity thresholds of *L. christinae* , enabling evidence‐based design of climate‐resilient conservation corridors. For instance, under the backdrop of climate change, key environmental factors can be improved through artificial interventions to promote the growth and spread of *L. christinae* . Specifically, vegetation restoration and ecological rehabilitation can be conducted in suitable areas to increase vegetation cover (Cai et al. 2022; Song et al. 2022), as well as improve soil and moisture conditions (Xu et al. 2021; Zhu et al. 2024), thereby enhancing the survival rate and reproductive capacity of *L. christinae* . The phenological characteristics of *L. christinae* , including its perennial growth habit and specific flowering period (April–June), may influence its response to climate change in ways not fully captured by annual climate variables. Additionally, the species' reliance on specific soil moisture conditions during its active growth phase and potential phenological mismatches with pollinators under altered climate regimes represent uncertainties in our projections. The model also does not account for the species' limited seed dispersal capacity (primarily gravity‐dispersed), which may constrain its ability to track suitable climate space. These biological constraints suggest that actual range shifts may lag behind climatic suitability shifts, particularly in fragmented landscapes.

### 4.3 Climate Change‐Driven Distributional Shifts of *L. christinae*

Across diverse climatic projections, the suitable habitats of *L. christinae* experienced marked shifts. Under the SSP1‐2.6 pathway, these areas displayed considerable consistency, maintaining preservation rates of 86.78%, 86.13%, and 82.03% in the 2050s, 2070s, and 2090s, respectively. This suggests that in a low‐emission context, the distribution of *L. christinae* was less affected, with limited contraction and expansion of suitable areas. Under the SSP3‐7.0 pathway, the retention rate of suitable areas dropped from 81.43% in the 2050s to 71.74% in the 2090s, indicating a notable tendency of reduction and expansion. During this period, the extent of marginally suitable regions exhibited a yearly rise, while regions of high suitability experienced a yearly decline. Under the SSP5‐8.5 pathway, the retention rate of suitable areas significantly decreased from 84.44% in the 2050s to 64.77% in the 2090s. This period experienced the greatest contraction and expansion of suitable areas, along with a notable increase in the area of generally suitable habitats and a corresponding decrease in the area of high suitability habitats. These changes reflect the profound impact of environmental change on the geographic occurrence of *L. christinae* , especially under high‐emission contexts, where the instability of suitable distribution areas increases, posing greater challenges to the species' survival and spread (Thuiller et al. 2008; Xian et al. 2023).

Compared to prior research (Xu, Lu, et al. 2024; Yu et al. 2023), this research more systematically analyzed the distribution changes at different future time points and under various climate scenarios, providing more detailed dynamic change data. Previous studies on medicinal herbaceous plants may have focused more on predicting distribution at current or single future time points (Guo et al. 2024; Zhang et al. 2023), whereas this investigation, through multi‐scenario comparisons, revealed the extended influence of global environmental variability on species' geographic spread. For instance, while some studies have only predicted distribution changes for the 2050s or 2090s (He, Si, et al. 2023; He, Ma, and Chen 2023), this study goes further. It predicts changes for the 2050s, 2070s, and 2090s and analyzes trends and characteristics under different climate scenarios. This approach provides a comprehensive understanding of how climate change affects species' ranges.

Our results provide crucial evidence for developing ecological conservation strategies for species in response to bioclimatic variability. For instance, drawing on predicted distribution trends, priority should be given to protecting areas with stable suitability or potential for expansion (Wu et al. 2023; Xia et al. 2022; Zou et al. 2023), enhancing ecological monitoring and management in these regions to ensure the survival and reproduction of *L. christinae* (Moore and Schindler 2022). Furthermore, the layout and scope of ecological reserves can be adjusted according to distribution trends (Liang et al. 2021; Schlaepfer and Lawler 2023), optimizing the allocation of ecological conservation resources and improving the efficiency and effectiveness of conservation efforts (Carroll and Ray 2021; Reside et al. 2018). These measures not only contribute to the protection of the rare plant resource *L. christinae* but also serve as a model for the preservation of other species, ensuring the stability and sustainable development of ecosystems (Dawson et al. 2011).

### 4.4 Limitations of the Study

Although the optimized MaxEnt model exhibited strong predictive power (AUC = 0.904), it is important to recognize several limitations. First, the occurrence data (*n* = 625), which were mainly sourced from herbarium collections and digital databases, might be biased toward regions that are more accessible, thereby potentially underrepresenting remote or less accessible habitats. Second, the 2.5′ resolution of the WorldClim variables may not be sufficient to capture the micro‐topographic refugia that are crucial for the survival of this species. Third, non‐climatic factors such as edaphic conditions, biotic interactions, and anthropogenic disturbances were not included in the model, despite their known importance in ecological contexts. Lastly, the model assumes that the species has full dispersal capability and does not account for potential migration barriers that could limit range shifts. Future research should consider integrating higher‐resolution environmental data, soil variables, and dispersal constraints to improve the accuracy of projections.

## 5 Conclusions

This study utilized an optimized MaxEnt modeling approach to assess the potential habitat shifts of *Lysimachia christinae*, a regionally endemic medicinal species in China, under multiple climate change scenarios. The model achieved an AUC value of 0.904, demonstrating robust predictive performance in characterizing the ecological niche of *L. christinae* . Our analysis identified the minimum temperature of the coldest month (Bio6) and the standard deviation of temperature seasonality (Bio4) as the primary climatic drivers, collectively explaining 88.9% of habitat suitability variations. These thermal variables play a critical role in determining the species' geographic range. Under the SSP1‐2.6 scenario, *L. christinae* exhibited minimal range contraction, with habitat retention rates of 86.8%, 86.1%, and 82.0% across the 2050s, 2070s, and 2090s, respectively, indicating relative stability in a low‐emission context. However, under the SSP5‐8.5 scenario, the retention rate of suitable habitats significantly declined to 64.77% by the 2090s, highlighting substantial habitat instability and the need for adaptive conservation strategies. These findings underscore the significant impact of climate change on the distribution of *L. christinae* , especially under high‐emission scenarios. Future research should focus on exploring additional environmental factors and conducting field studies to validate model predictions and enhance conservation efforts.

## Author Contributions

**Yangzhou Xiang:** funding acquisition (equal), methodology (equal), visualization (equal), writing – original draft (equal), writing – review and editing (equal). **Yuan Li:** conceptualization (equal), methodology (equal), writing – original draft (equal), writing – review and editing (equal). **Ying Liu:** data curation (equal), funding acquisition (equal), software (equal). **Yingying Yuan:** data curation (equal), software (equal). **Suhang Li:** data curation (equal), software (equal), visualization (equal). **Qiong Yang:** data curation (equal), software (equal), visualization (equal). **Jinxin Zhang:** conceptualization (equal), funding acquisition (equal), project administration (equal), writing – review and editing (equal).

## Conflicts of Interest

The authors declare no conflicts of interest.

## Supporting information


Appendix S1.

## Data Availability

Location records and environmental variables have been uploaded to an open data repository via Figshare (https://figshare.com/articles/dataset/Dataset/29234846).
