# Supplementary material for: Assessing Climate Change Impacts on Distribution Dynamics of Lysimachia Christinae in China Through MaxEnt Modeling
Source: Ecol Evol. 2025 Jun 24;15(6):e71664. doi: 10.1002/ece3.71664 (PMC12185935; doi:10.1002/ece3.71664)
Supplement: Supplementary file 1 — Appendix S1. [file ECE3-15-e71664-s001.docx]

**Supporting Information**

**Assessing Climate Change Impacts on Distribution Dynamics of *Lysimachia christinae* in China Through MaxEnt Modeling**

Yangzhou Xiang ^1^, Yuan Li ^2^, Ying Liu ^3^, Yingying Yuan ^1^, Suhang Li ^1^, Qiong Yang ^1^, and Jinxin Zhang ^4,^ *

^1^ School of Geography and Resources, Guizhou Education University, Guiyang 550018, China

^2^ Grasslands and Sustainable Farming, Production Systems Unit, Natural Resources Institute Finland, Halolantie 31A, Maaninka, Kuopio FI-71750, Finland

^3^ School of Biological Sciences, Guizhou Education University, Guiyang 550018, China

^4^ Institute of Ecological Conservation and Restoration, Chinese Academy of Forestry/Grassland Research Center, National Forestry and Grassland Administration, Beijing 100091, China

* Correspondence: zhangjinxin@caf.ac.cn (J.Z.)

**Table S1**. Nineteen environmental variables used in this study.

| Variables | Description | Units | Range | Contribution rate (%) |
| --- | --- | --- | --- | --- |
| Bio1 | Annual mean temperature | °C | -0.4－22.3 | 0 |
| Bio2 | Mean diurnal range (Mean of monthly) | °C | 6.0－13.1 | 1.0 |
| Bio3 | Isothermality (Bio2/Bio7) (× 100) |  | 22.7－48.0 | 1.7 |
| Bio4 | Standard deviation of temperature seasonality |  | 414.6－969.4 | 5.0 |
| Bio5 | Max temperature of warmest month | °C | 12.7－34.3 | 0.3 |
| Bio6 | Min temperature of coldest month | °C | -17.7－9.8 | 34.2 |
| Bio7 | Temperature annual range (Bio5-Bio6) | °C | 18.8－37.1 | 2.3 |
| Bio8 | Mean temperature of wettest quarter | °C | 7.0－28.7 | 0.2 |
| Bio9 | Mean temperature of driest quarter | °C | -8.9－18.1 | 4.5 |
| Bio10 | Mean temperature of warmest quarter | °C | 7.0－28.7 | 1.1 |
| Bio11 | Mean temperature of coldest quarter | °C | -8.9－14.7 | 0.9 |
| Bio12 | Annual precipitation | mm | 553.0－2130.0 | 45.7 |
| Bio13 | Precipitation of wettest month | mm | 106.0－422.0 | 0.1 |
| Bio14 | Precipitation of driest month | mm | 1.0－51.0 | 0 |
| Bio15 | Variation of precipitation seasonality |  | 39.7－102.1 | 0.4 |
| Bio16 | Precipitation of wettest quarter | mm | 286.0－985.0 | 0 |
| Bio17 | Precipitation of driest quarter | mm | 8.0－197.0 | 0 |
| Bio18 | Precipitation of warmest quarter | mm | 249.0－963.0 | 0.8 |
| Bio19 | Precipitation of coldest quarter | mm | 8.0－240.0 | 1.6 |
